# P-407. Evaluation of Use of Hospital-onset Bacteremia and Fungemia (HOB) for Quality Improvement

**DOI:** 10.1093/ofid/ofae631.608

**Published:** 2025-01-29

**Authors:** Gregory M Schrank, Surbhi Leekha, Mya Brady, Amber R Thomas, Ayda Soltanian Tiranchi, Gwen Robinson, Elise Martin, Graham M Snyder

**Affiliations:** University of Maryland School of Medicine, Baltimore, Maryland; University of Maryland School of Medicine, Baltimore, Maryland; University of Pittsburgh Medical Center, Pittsburgh, Pennsylvania; University of Maryland Medical Center, Baltimore, Maryland; University of Maryland School of Medicine, Baltimore, Maryland; University of Maryland, Baltimore, Baltimore, Maryland; VA Pittsburgh Healthcare System, Pittsburgh, Pennsylvania; University of Pittsburgh, Pittsburgh, PA

## Abstract

**Background:**

Hospital-onset bacteremia and fungemia (HOB) is under development as a surveillance measure by the CDC. This study aimed to evaluate the use of HOB by frontline clinicians to identify infection prevention (IP) and quality improvement opportunities.

**Figure 1:**
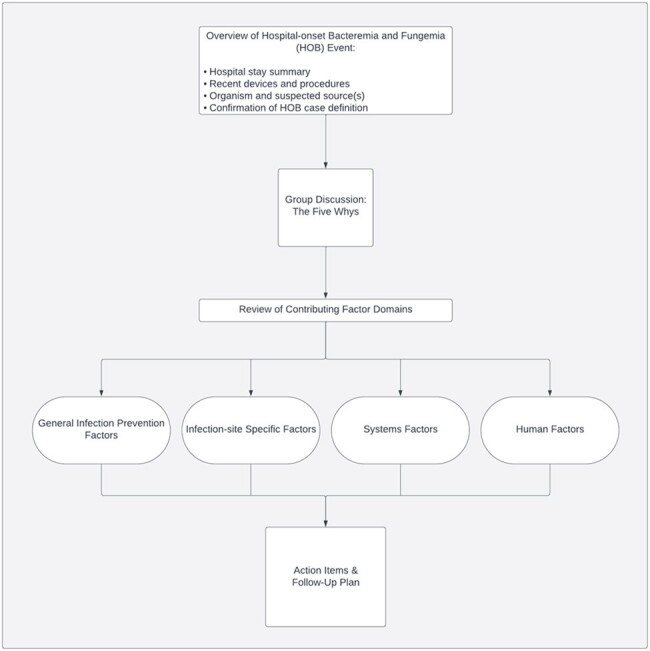
Framework for evaluation of hospital-onset bacteremia and fungemia cases with multi-disciplinary case reviews, adapted from the Joint Commission Patient Safety Event Taxonomy.

**Methods:**

From 9/2022 to 4/2024, we conducted a prospective study at 6 hospitals (3 academic, 3 community) affiliated with two academic health systems. We adapted the Joint Commission Patient Safety Event Taxonomy to develop a framework for review of HOB events. (Figure 1). Within four primary domains of general IP, infection-site-specific, system, and human factors, cascading questions elicited potential contributing causes. We used the “Five Whys” technique to guide multidisciplinary case reviews and refined the framework using feedback from the first 15 reviews. HOB events identified from existing hospital databases were selected using convenience sampling, intentionally limiting inclusion of events associated with neutropenia or central line infections. Virtual case reviews were conducted within 4 weeks of the event. We summarized HOB sources, contributing factors, and improvement opportunities, using descriptive analyses.

**Figure:**
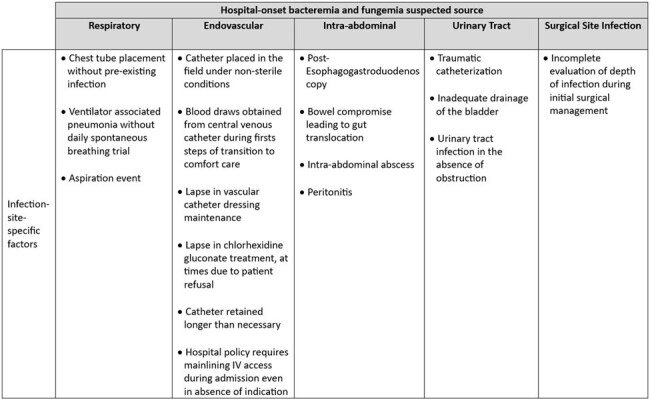

Infection-site-specific factors identified during case reviews as contributing to the most common hospital-onset bacteremia and fungemia sources.

**Results:**

75 HOB events were discussed by a median of 3 participants per review. The most common suspected sources of HOB were respiratory (21%), endovascular (20%), intra-abdominal (17%), urinary tract (15%), and surgical site infection (9%); 25% were of unknown source. IP factors were identified in 51% HOB events and included challenges with hand hygiene (40%), equipment and environmental cleaning (12%), personal protective equipment compliance (5%), and multi-drug resistant organism transmission (3%). Infection-site-specific factors were identified in 36 (48%) of HOB events (Table 1). System factors and human factors were identified in 59% and 32%, respectively (Table 2). Select HOB cases and action items determined from case reviews are listed in Table 3.

**Figure:**
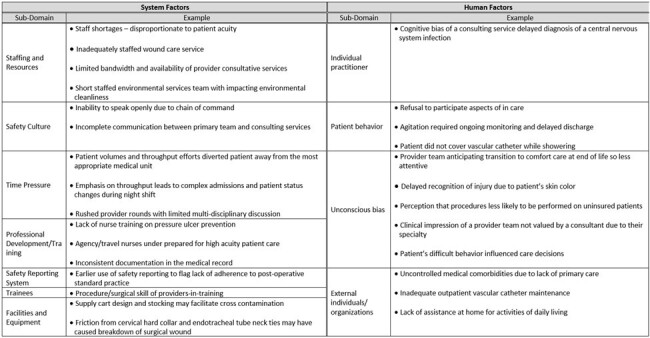

Examples of system and human factors contributing to hospital-onset bacteremia and fungemia events elicited during case reviews, organized by sub-domain.

**Conclusion:**

HOB events have diverse causes and elements of preventability, and they can be used as sentinel events to guide quality of care review. HOB sources and contributing factors beyond the typical focus of IP can provide insight into preventability beyond guideline-defined measures.

**Figure:**
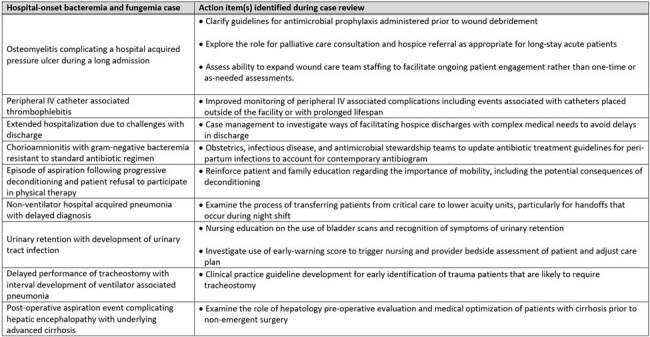

Select hospital-onset bacteremia and fungemia cases and action items for patient safety/quality improvement identified during multi-disciplinary case reviews.

**Disclosures:**

**Gregory M. Schrank, MD MPH**, EnSenSys, LLC: Grant/Research Support **Graham M. Snyder, MD, SM**, Infectious Diseases Connect: Advisor/Consultant

